# Influence of salinity and temperature on uptake of perfluorinated carboxylic acids (PFCAs) by hydroponically grown wheat (*Triticum aestivum L.*)

**DOI:** 10.1186/s40064-016-2016-9

**Published:** 2016-04-27

**Authors:** Hongxia Zhao, Baocheng Qu, Yue Guan, Jingqiu Jiang, Xiuying Chen

**Affiliations:** Key Laboratory of Industrial Ecology and Environmental Engineering (Ministry of Education), School of Environmental Science and Technology, Dalian University of Technology, Linggong Road 2, Dalian, 116024 China; Dalian Institute of Food Inspection, Dalian, 116630 China

**Keywords:** Perfluoroalkyl substances (PFASs), Wheat, Salinity, Temperature, Transfer factor (TF)

## Abstract

Perfluoroalkyl substances (PFASs) have recently attracted increasing concerns due to their ubiquitous existence, adverse effects and persistence in environment. This study employed four perfluorinated carboxylic acids (PFCAs) to examine effects of salinity and temperature on the PFAS uptake in wheat, one of the major crops in the North China Plain. Wheat plants were grown in the spiked-PFCA hydroponic culture system at different salinities and temperatures. As expected, salinity and temperature significantly impacted the root uptake and translocation of wheat to four PFCAs, and the concentrations for each of PFCAs in wheat root and shoot increased with increasing salinity and temperature, respectively. PFCA concentrations at high salinity or high temperature were up to thrice those found at low salinity or low temperature. Except for perfluorobutanoic acid, the amount of PFCAs in root was always higher than that in shoot at the ranges of salinity and temperature tested. Additionally salinity and temperature were also capable of influencing the transfer factors (TFs) of four PFCAs, and significant increase was observed in the TFs in response to the increases in salinity and temperature.

**Electronic supplementary material:**

The online version of this article (doi:10.1186/s40064-016-2016-9) contains supplementary material, which is available to authorized users.

## Background

Perfluoroalkyl substances (PFASs) are a group of manmade organic chemicals which have been considerably produced and applied as surfactants and surface protectors in leather, paper, packaging, and upholstery, and in or as aqueous film fire-fighting foams, mining and oil well surfactants, shampoos, and insecticide for more than 50 years (OECD 2002; Lau et al. 2007). Industrial and commercial PFASs contain a large number of compounds such as perfluorinated carboxylic acids (PFCAs), perfluorinated sulfonic acids (PFSAs) and their precursors—fluorotelomer acids, fluorotelomer alcohols, etc. (Murphy et al. 2012). The global annual production volume of perfluorooctanoic acid (PFOA) was 1200 t in 2004, and perflurooctane sulfonate (PFOS) production reached 3500 t only in 2000 (Lau et al. 2007). As a result of their extensive production and long-term use, PFASs have been globally detected in a variety of biotic and abiotic matrices including mussel (Renzi et al. 2013), macroalgae (Kelly et al. 2009), fish (Sinclair et al. 2006), breast milk (Mondal et al. 2014), sediment (Corsolini et al. 2012), surface water (Rostkowski et al. 2009), groundwater (Eschauzier et al. 2013), and rain (Lin et al. 2014), etc. In addition to their ubiquity, these chemicals were also found to have moderate acute toxicity, and some animal studies have shown that exposure to PFASs may result in hepatotoxicity, neurotoxicity, immunotoxicity, developmental toxicity and cancer (Joensen et al. 2009; Hoffman et al. 2010; Melzer et al. 2010; Steenland et al. 2010; Kim et al. 2011). Thus, as the most important PFASs, PFOS has been listed as POPs in Annex B of the Stockholm Convention in 2009 (http://chm.pops.int/TheConvention/ThePOPs/ListingofPOPs/tabid/2509/Default.aspx) and PFOA, PFOS, as well as their precursors have been recommended monitoring of the presence in food by EU in 2010 (http://eur-lex.europa.eu/LexUriServ/LexUriServ.do?uri=OJ:L:2010:068:0022:0023:EN:PDF).

In recent years, there is a growing concern of human exposure pathways to PFASs due to their high concentrations found in human samples (Nilsson et al. 2010; Mondal et al. 2014). Besides household dust (Haug et al. 2011) and drinking water (Eschauzier et al. 2013), consumed foodstuffs have been proved to be the most important source of PFAS intake for non-occupationally exposed humans (Domingo et al. 2012). A recent study in Sweden has confirmed the existence of a strong correlation between PFAS levels in human blood serum and food consumption (Bjermo et al. 2013). Therefore, more information is needed on PFAS contamination pathways to food, which is very important to assess the risk of potential exposure of human to these compounds.

As one of the basic diets for human and animals, plants may be one way of entry of PFASs into human. Market basket studies have shown that some plant food items contain high levels of PFASs. Plant exposure to PFASs may be mainly via plant root uptake from the contaminated soils or dry deposition from the atmosphere to the above-ground parts. Until now, only a few studies have been performed to investigate PFAS uptake by plant. Garcia-Valcarcel et al. (2014) studied uptake of PFCs by a grass (*B. diandrus*) grown in nutrient solution, and assessed the rate and extent of PFAS uptake by plant. Felizeter et al. (2012) investigated root uptake efficiency and distribution of 14 PFASs in hydroponically grown lettuce (*Lactuca sativa*) and found the uptake by root was governed by sorption of PFASs to lipid-rich root solid and their translocation from root to shoot which highly depend on the hydrophobicity of the compounds. Stahl et al. (2009) and Lechner and Knapp (2011) reported that PFOS and PFOA were able to be transferred from contaminated soils to plants including rye grass, potatoes, carrots and cucumbers, and accumulation occurred much less in storage organs (e.g., tubers, ears) of the plants than in vegetative compartments (e.g., straw, leaves). Additionally, Yoo et al. (2011) also observed linear relationships between log gras-accumulation factors for C_6_–C_14_ PFCAs and carbon chain length in their uptake study about grass species. To sum up, these data are important for us to understand the exposure pathways of PFASs to plants.

Plant uptake is generally dependent on the physical–chemical properties of the chemicals, the characteristics of soil and irrigation water, and the plant species and physiology (Paterson et al. 1994). It has been known that the changes in environmental influences (such as salinity and temperature) may impact the physicochemical properties of compounds as well as the physiology of plants, consequently resulting in changes in contaminant uptake by plant (Stofberg et al. 2015). To date, the uptake potential of PFASs by plant in salinity or temperature gradients is unclear. The purpose of this study is to examine the effects of salinity and temperature on the PFAS uptake by root, and their subsequent translocation to shoot in wheat. Four PFCAs including shorter chain perflurobutanoic acid (PFBA), long chain perfluoroheptanoic acid (PFHpA), PFOA and longer chain perfluorododecanoic acid (PFDoA) were selected as model compounds and their uptakes in wheat at different salinities and temperatures were evaluated in a hydroponic culture system which is easy to control the exposure conditions and avoid the differences caused by the varied adsorption of PFCAs to soil.

## Methods

### Materials

PFBA, 99 %, PFHpA, 98 %, and PFDoA, 96 % were all obtained from Sigma Aldrich (Zwijndrecht, Netherlands). PFOA, 98 %, was purchased from Fluka (Steinheim, Switzerland). The stock solutions of testing chemicals (1 mg/mL) were prepared in the deionized water from a Milli-Q water purification system (Millipore, Milford, MA) and stored in polymethyl pentene containers. All organic solvents for extraction and cleanup were of HPLC grade and the reagents for preparing the Hoagland solution were analytical grade. The variety of tested wheat (*T. aestivum* L.) was Liaoning Spring No. 10, which was bought from Liaoning Dongya Seed Co. in Shenyang, China.

### Plant culture and exposure experiments

The wheat culture has been introduced in our previous study (Zhao et al. 2013). In brief, the wheat seeds with sterilization treatment were germinated on wet filter paper for 7 days, and then fifty seedlings were transferred to 1 L glass beakers containing half-concentrated Hoagland’s solution (Lin and Xing 2008) for further growth at 25 °C and a lighting period of 16 h per day. When their roots reached 8 ± 1 cm, and leaves grew 10 ± 1 cm, these seedlings were transferred to an exposure apparatus containing half-concentrated Hoagland’s solution with each of PFCAs at 1 μg/mL for the study of PFCA uptake induced by salinity and temperature. After the wheat was grown for five days in nutrient solution at different salinities (0.1, 0.2, 0.3 and 0.4 %) or different temperatures (20, 25 and 30 °C), they were harvested at a time. The harvested wheat was divided into shoots and roots which were washed in deionized water to remove any external salt or PFCA residues and patted dry with a paper towel, and stored at −20 °C until extraction. Three replicates for each salinity and temperature were used, including blank controls. During the five days of exposure, the PFCA-spiked nutrient solution was exchanged three times to keep the PFCA’s concentration at a constant level and the PFCA concentrations in the nutrient solution were also monitored.

### Sample extraction and analysis

The extraction procedure was similar to that described elsewhere with minor modifications (Shi et al. 2010). In brief, after roots or shoots were homogenized with household blender, 0.1 g (fresh weight) of the homogenate was put into a 10 mL polypropylene tube, then 1 mL of 170 g/L tetrabutyl ammonium hydrogen sulfate solution and 2 mL of 26.5 g/L sodium carbonate buffer were added for extraction. After mixing, 4 mL of methyl-*tert*-butyl ether (MTBE) was added, and the mixture was shaken for 20 min. The organic and aqueous layers were separated by centrifugation (12,000×*g*), and the organic solvent was transferred from the solution. The aqueous mixture was rinsed with MTBE and separated twice. The combined organic solvent was allowed to evaporate to dry under nitrogen at about 50 °C before being reconstituted in 1.5 mL of methanol. The sample was vortexed for 30 s and passed through a 0.2 μm nylon mesh filter into an auto sampler glass vial.

PFCAs were analyzed by Agilent 1200 HPLC coupled to a 6410 Triple Quadrupole mass spectrometer (Agilent Technologies, Santa Clara, CA) equipped with an electrospray source operating in negative ionization mode. A 5 μL aliquot of extract was injected onto an Agilent Eclipse Plus C18 column (2.1 mm i.d. × 100 mm length, 3.5 μm) (Agilent Technologies, Palo Alto, CA) with 10 mM ammonium acetate/water and acetonitrile as mobile phases at a flow rate of 0.25 mL/min. Column temperature was maintained at 40 °C. The gas temperature and ion spray voltage were maintained at 350 °C and 4000 V. Ions were monitored with a multiple reaction monitoring (MRM) mode, and the parameters for parent and product ions and collision energies of each target analyte were listed in Additional file 1: Table S1. Each of PFCAs was quantitated by external standard calibration curve and the peak area counts from the chromatogram, and the standard calibration curves were shown in Additional file 1: Figure S1.

### Quality assurance and quality control

The procedural blanks were determined simultaneously for each batch of three samples by going through the same extraction and cleanup methods as the root samples or the shoot samples from plants growing in nonspiked nutrient solution. PFCAs except for PFBA were detected in roots, and the background concentration of PFDoA was always above the detection limit. PFBA and PFHpA were found in shoots, and the background concentration in shoot above the detection limit was observed for PFBA. The resulting concentrations were calculated by subtracting background concentrations from the concentrations determined in the spiked nutrient solutions. The method accuracy was evaluated by analyzing eight replicates on the basis of repeatability and the obtained relative standard deviations (RSD) were 6.5–13.6 % in root sample and 7.8–15.4 % in shoot sample. Recoveries were evaluated by spiking blanks with a low concentration standards of four PFCAs and were found to be 68.3–91.5 % in root sample and 60.5–84.6 % in shoot sample, respectively (n = 10).The limit of detection (LOD) was defined as the concentration that would result in a signal-to-noise ratio of 3, and the LODs for four PFCAs were determined in the light of the extracted wet roots or shoot mass (0.5 g), and instrumental sensitivity. The LODs in root samples ranged from 0.02 to 0.06 ng/g (fresh weight) and the ones in shoot samples were in the range of 0.04–0.07 ng/g (fresh weight).

### Data handling

Statistical analysis was performed using the SPSS (version 13.0, SPSS Inc. 2004). A probability value p < 0.05 was thought as statistically significant. A one-way analysis of covariance (ANCOVA) was used to test the significance of differences among means. Linear least squares regression was used to describe the relationship between the concentrations in the above-ground parts and in the roots. Data are presented as the means (±standard error) of three replicates.

## Results and discussion

### Effects of PFCAs, salinity and temperature on growth rate and biomass of wheat

Neither mortality nor plant health (spots, decoloring) was observed in the control group and the treated groups during the whole test period. Two morphological indices, growth rate and biomass, were used to evaluate the overall effects of PFCAs, salinity and temperature on wheat seedlings. Growth rate was measured as increase in length (cm/5 day) and biomass was determined as increase in dry weight (g/5 day). Slightly higher growth rates were found in wheat exposed to four PFCAs at concentration of 1 μg/mL than in the control group (see Additional file 1: Table S2), and the average dry weight for wheat exposed to four PFCAs was observed with a 2.1 % increase as compared to the controls. These findings indicated four PFCAs had no obvious inhibition in the growth of wheat at the studied concentration of 1 μg/mL. Growth rates small increased with increasing temperature from 20 to 30 °C, but the dry weights did not differ significantly among treatments (p < 0.05). No significant differences (p < 0.05) in growth rates or dry weights were found among salinity treatments. As experimental parameters were similar for all conditions throughout the experiment, the obtained results of PFCA uptake in wheat could be compared.

### Uptake of PFCAs by wheat at different salinities and temperatures

**Fig. 1:**
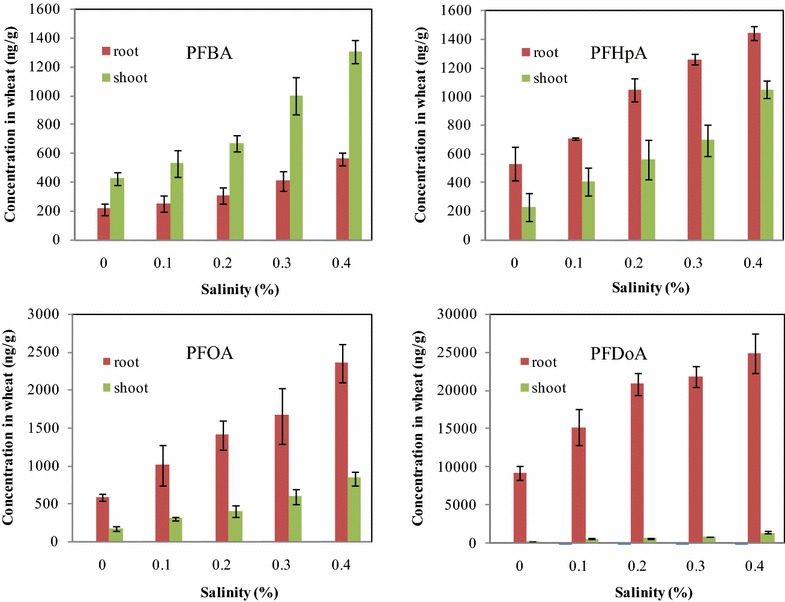
The concentrations of PFCAs taken up by the roots and shoot of wheat seedling at different salinity conditions

**Fig. 2:**
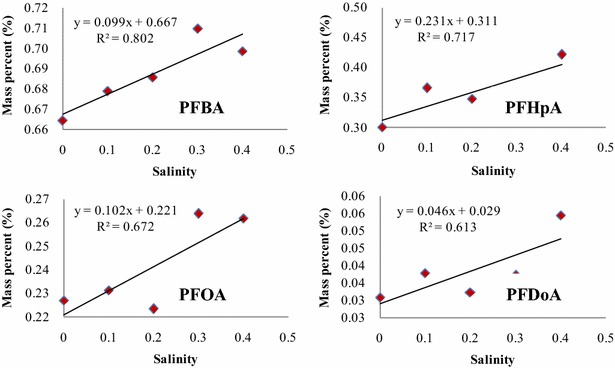
The positive correlation between the mass percent of PFCAs in the above-ground parts and salinity

The background concentrations of PFCAs in the control wheat were below the detection limits, 0.02–0.07 ng/g fresh weight, except for PFBA, 1.8 ng/g fresh weight in shoots, and PFDoA, 3.6 ng/g fresh weight in roots. The results of the recovery tests with fortified wheat samples are reported in supporting information (Additional file 1: Table S3). Uptake of PFCAs in wheat exposed to four different salinities is depicted in Fig. 1. As shown in Fig. 1, the concentrations of PFCAs in wheat increased in response to the increase of salinity and the highest concentrations in wheat root and shoot were found to be in the range of 1870.8–26287.2 ng/g for four PFCAs at the highest salinity treatment. The uptake of PFCAs increased by 2.9-fold for PFBA, 3.3-fold for PFHpA, 4.2-fold for PFOA, and 2.8-fold for PFDoA as salinity increased from 0 to 0.4 %, and the positive linear relationships were found between the increases in salinity and in amounts of each of PFCAs absorbed by wheat plant with correlation coefficient square higher than 0.84 (Additional file 1: Figure S2). Among the four PFCAs tested, PFDoA was the most accumulative in wheat, whereas PFBA was the least. The significant concentration gradients (p < 0.05) were observed for four PFCAs in the salinity range tested, and for PFHpA, PFOA and PFDoA, the high concentrations were found in the roots and the low in the shoots, whereas for PFBA, the concentration in the shoots was higher than that in the roots. The similar results were also reported in previous studies (Stahl et al. 2009; Felizeter et al. 2012; Garcia-Valcarcel et al. 2014). To further assess the partitioning of four PFCAs within wheat at different salinities, the relationships between PFCA concentrations in the roots and in the aerial parts were established, and the significant linear relationships were found between concentrations of individual PFCAs in the aerial parts and in the roots (p < 0.05) (Additional file 1: Figure S3). The mass percent content for each of PFCAs distributed in the aerial parts was further calculated (Additional file 1: Table S4), and the mass percent of PFCAs in the aerial parts increased with increasing salinity (Fig. 2). These results indicated the increase of salinity was favorable to the uptake of PFCAs in wheat grown in a hydroponic system.

Salinity has been shown to affect the physicochemical properties of chemicals as well as the physiology of organisms, consequently leading to changes in contaminant uptake and toxicity (Dyer et al. 1989; Hall and Anderson 1995). In general, plant grown at high salinity (>0.2 %) can decrease their water availability for transpiration and the osmotic head in the root zone, inevitably accompanied by a decrease in the uptake of contaminants. In this study, when salinity was enhanced from 0.2 to 0.4 %, the uptake of four PFCAs by wheat still increased (Fig. 1; Additional file 1: Figure S2). According to previous reports (Ungar 1996; Jeon et al. 2010; You et al. 2010), we assumed this may be caused by two reasons: (1) with increasing salinity, to maintain proper osmotic pressure, wheat increases water uptake, and, therefore, the PFCA uptake is increased; (2) with increasing salinity, the ion strength of solution increases, which may lead to a higher absorption of PFCAs to wheat root, and then increase the PFCA uptake.

**Fig. 3:**
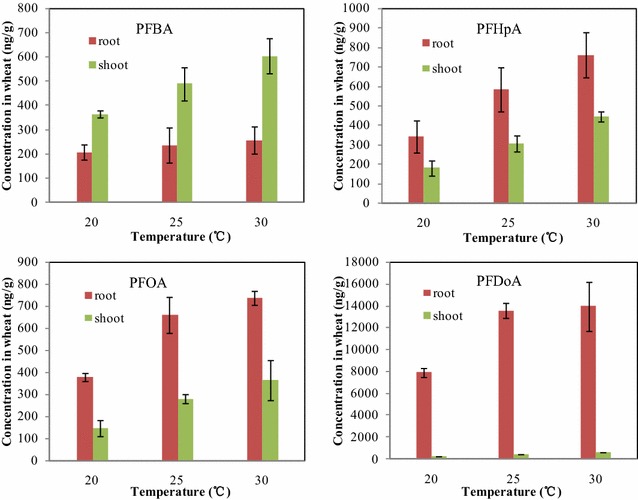
The concentrations of PFCAs taken up by the roots and shoot of wheat seedling at different temperature conditions

Three different growth temperatures of 20, 25 and 30 °C were selected to study temperature effect on PFCA uptake in wheat. The results are represented in Fig. 3. As shown in Fig. 3, the concentrations of PFCAs in wheat increased with the increase of temperature. The highest concentrations in the roots and shoots of wheat were found to be 858 ng/g for PFBA, 1205 ng/g for PFHpA, 1098 ng/g for PFOA and 14576 ng/g for PFDoA at the highest exposure temperature of 30 °C and the wheat uptake to PFCAs increased by 1.5–2.3-fold as temperature increased from 20 to 30 °C, and the linear regression analysis showed that the increases for four PFCAs in concentration and temperature were statistically significant at p < 0.05 with correlation coefficient square greater than 0.90 in the temperature range tested (Additional file 1: Figure S4). The similar concentration gradients were also found for four PFCAs and except for PFBA, the higher concentrations were always determined in the roots than in the shoots for the other three PFCAs. The mass percent contents of four PFCAs in the aerial parts were also calculated individually (Additional file 1: Table S4), and the mass percent of four PFCAs in the aerial parts increased with the increase of temperature (Additional file 1: Figure S5). These results showed the increase of temperature was also favorable to the uptake of PFCAs in wheat.

Temperature is an important parameter for plant growth, which may indirectly affect plant uptake to chemical via influencing water and nutrient uptake, and metabolic processes (Dong et al. 2001). In general, when plants are grown at their optimum temperature range, the increase of the growth temperature can enhance the diffusion rate of nutrient, decrease the viscosity of water, and provide more energy for the photosynthesis, consequently followed by an increase in the uptake of contaminants. Hence, this study provides a direct evidence under controlled conditions that uptake of PFCAs by wheat is favored by increasing temperature. In addition, we found that the concentrations of longer-chain PFCAs in wheat such as PFDoA increased more rapidly with temperature compared to those of shorter-chain PFCAs such as PFBA, and the C_high temperature_/C_low temperature_ ratio (slope of the lines in Additional file 1: Figure S4) for PFDoA was more than twenty times higher than that for PFBA, which indicated that the uptake of wheat to longer-chain PFCAs might be more sensitive to temperature than to shorter-chain PFCAs (Additional file 1: Table S5).

### Effects of salinity and temperature on the transfer factors

**Fig. 4:**
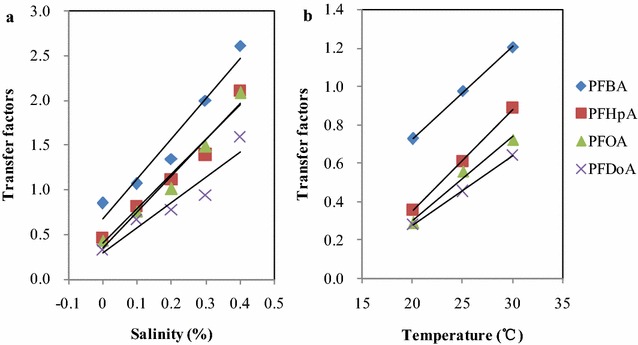
Effects of salinity and temperature on the transfer factors (TFs) of PFCAs in wheat **a** salinity, **b** temperature

Transfer factors (TF) are defined as the ratio of the concentration of a compound in aerial tissues (ng/g fresh plant) divided by its concentration in the nutrient solution (ng/mL) or soil, which are generally used to evaluate the transport of a compound from solution or soil to the aerial parts. The transfer factors for each of the PFCAs at different exposure salinity and temperature conditions were calculated and presented in Fig. 4 and Additional file 1: Table S5. As Fig. 4 and Additional file 1: Table S5 shown, the transfer factors of PFASs were 0.85 for PFBA, 0.46 for PFHpA, 0.43 for PFOA, and 0.33 for PFDoA in the control wheat which was exposed to the PFCA-spiked solution without addition of NaCl, and the transfer factors significantly decreased with carbon chain length (p < 0.05). The TF values of PFOA obtained in the control wheat were basically in agreement with a previous study (Lechner and Knapp 2011) and the similar change trend was also found by Yoo et al. (2011) and Garcia-Valcarcel et al. (2014) who reported that the shortest perfluorinated carboxylates had the highest transfer factor decreasing over the homologue range. Salinity has a significant effect on the TF values of four PFCs and as Fig. 4a shown, the TF values increased with the increase of salinity. The highest TF values for four PFCAs were obtained for plant growing in the solution with the highest salinity treatment. Compared to the control, the TF values for four PFCAs at the highest salinity were correspondingly 3.1–4.9 times higher than those in the control. This can be possibly explained by higher uptake amount in wheat evoked by salinity than that in the control.

Temperature can significantly impact the transfer factors of PFCAs in wheat (p < 0.05) and as shown in Fig. 4b, with increasing temperature, the TF values significantly increased. The lowest TF values for four PFCAs were found in plant growing in the solution at 20 °C, and they were 0.73, 0.36, 0.29 and 0.28 for PFBA, PFHpA, PFOA, PFDoA, respectively. When exposure temperature was enhanced to 30 °C, the TF values for four PFCAs increased correspondingly by 1.7–2.5 times compared to those at 20 °C, which was possibly attributed to the increase of uptake amount induced by high temperature.

## Conclusion

In this study, we investigated the influence of salinity and temperature on uptake of PFCAs by wheat. Our results showed both salinity and temperature were capable of inducing the increase of PFCA uptake and translocation in wheat grown in a hydroponic culture system. The TF values of four PFCAs were enhanced by 1.7–4.9 times as initial salinity increased from 0 to 0.4 % and growth temperature increased from 20 to 30 °C. These findings indicate the changes in salinity and temperature should be taken into consideration to assess exposure risks of PFCAs to terrestrial organism including human. In addition, given that a competing hydrophobic interaction caused by organic carbon in soil may reduce the transfer rate of PFAS to roots, it is very necessary to further investigate the uptake of PFCAs by plant in more environmentally relevant systems, e.g. by using natural organic matter.

## Additional file

10.1186/s40064-016-2016-9 PFCAs’ linear relationships. **Fig. S1.** Standard calibration curves; **Fig. S2.** Between the absorbed PFCAs by wheat and the added salinity; **Fig. S3.** Between the concentrations of individual PFCAs in the aboveground parts and in the roots; **Fig. S4.** Between the absorbed PFCAs by wheat and temperature; **Fig. S5**. Between the mass percent of PFCAs in the above-ground parts and temperature. PFCA’s analytical parameters. **Table S1.** The MS/MS parameters; **Table S2.** The root lengths and dry weight; **Table S3.** The recoveries; **Table S4.** The mass percent composition; **Table S5.** The transfer factors.
