# Supplementary material for: Influence of salinity and temperature on uptake of perfluorinated carboxylic acids (PFCAs) by hydroponically grown wheat (Triticum aestivum L.)
Source: Springerplus. 2016 Apr 27;5:541. doi: 10.1186/s40064-016-2016-9 (PMC4846607; doi:10.1186/s40064-016-2016-9)
Supplement: Supplementary file 1 — 10.1186/s40064-016-2016-9 PFCAs’ linear relationships. Fig. S1. Standard calibration curves; Fig. S2. Between the absorbed PFCAs by wheat and the added salinity; Fig. S3. Between the concentrations of individual PFCAs in the aboveground parts and in the roots; Fig. S4. Between the absorbed PFCAs by wheat and temperature; Fig. S5. Between the mass percent of PFCAs in the above-ground parts and temperature. PFCA’s analytical parameters. Table S1. The MS/MS parameters; Table S2. The root lengths and dry weight; Table S3. The recoveries; Table S4. The mass percent composition; Table S5. The transfer factors. [file 40064_2016_2016_MOESM1_ESM.docx]

**Supporting Information**

**Influence of salinity and temperature on uptake of** **perfluorinated carboxylic acids(PFCAs)by hydroponically grown wheat (*Triticum aestivum L.*)**

Hongxia Zhao^1*^, Baocheng Qu^2^, Yue Guan^1^, Jingqiu Jiang^1^, Xiuying Chen^1^

^1^Key Laboratory of Industrial Ecology and Environmental Engineering (Ministry of Education), School of Environmental Science and Technology, Dalian University of Technology, Linggong Road 2, Dalian 116024, China

^2^Dalian Institute of Food Inspection, Dalian 116630, China

Corresponding author phone: +86-411-84707965; Fax: +86-411-84707965; Email: [hxzhao@dlut.edu.cn](mailto:hxzhao@dlut.edu.cn)

Figure S1-S5·························································································Page S2-S4

Table S1-S4··························································································Page S5-S6

Figure S1 The standard calibration curves of four PFCAs

Figure S2 The linear relationship between the absorbed PFCAs by wheat and the added salinity

Figure S3The correlation between the concentrations of individual PFCAs in the aboveground parts and in the roots

Figure S4 The linear relationship between the absorbed PFCAs by wheat and temperature

Figure S5 The positive correlation between the mass percent of PFCAs inthe above-ground parts and temperature

Table S1 the MS/MS parameters of the method

| **Compound** | **Parent ions** | **Product ions** | **Collision energies** | **Retention time (min)** |
| --- | --- | --- | --- | --- |
| **PFBA** | 213 | 169 | 5 | 6.5 |
| **PFHpA** | 363 | 319,169 | 5,10 | 7.1 |
| **PFOA** | 413 | 369,169 | 5,15 | 9.2 |
| **PFDoA** | 613 | 569,319 | 5,10 | 12.1 |

Table S2the root lengths and dry weight of wheat for exposed PFCAs and control wheat

|  | Number of wheat | Uptake period (d) | Mean root  length after 5 d (cm) | Mean shoot  length after 5 d(cm) | Mean dry root weight after 5 d (g) | Mean dry shoot weight after 5 d (g) |
| --- | --- | --- | --- | --- | --- | --- |
| Control | 50 | 5 | 7.6±0.5 | 10.9±0.6 | 0.24±0.1 | 0.60±0.1 |
| PFBA  -exposed wheat | 50 | 5 | 7.8±0.4 | 11.3±0.3 | 0.25±0.1 | 0.60±0.1 |
| PFHpA -exposed wheat | 50 | 5 | 7.9±0.5 | 11.2±0.4 | 0.26±0.1 | 0.60±0.1 |
| PFOA  -exposed wheat | 50 | 5 | 8.0±0.4 | 11.4±0.4 | 0.27±0.1 | 0.60±0.1 |
| PFDoA -exposed wheat | 50 | 5 | 7.8±0.5 | 11.1±0.5 | 0.25±0.1 | 0.60±0.1 |

Table S3 the recoveries of four PFCAs spiked in the roots and shoots of wheat

| **Compound** | **Spiked/(ng/L)** | **Root (n=3)** | | **Shoot (n=3)** | |
| --- | --- | --- | --- | --- | --- |
|  |  | **Mean/%** | **RSD/%** | **Mean/%** | **RSD/%** |
| **PFBA** | 2 | 68.3 | 13.6 | 60.5 | 2.9 |
|  | 20 | 72.9 | 4.1 | 68.1 | 11.2 |
| **PFHpA** | 2 | 75.0 | 5.0 | 73.2 | 9.3 |
|  | 20 | 84.5 | 3.1 | 78.8 | 9.7 |
| **PFOA** | 2 | 77.6 | 11.5 | 74.1 | 13.9 |
|  | 20 | 80.8 | 5.5 | 80.1 | 11.5 |
| **PFDoA** | 2 | 84.1 | 10.9 | 77.3 | 9.6 |
|  | 20 | 91.5 | 6.5 | 84.6 | 7.1 |

Table S4The mass percent composition for PFCAsdistributed in the above-ground parts at different salinity and temperature

| **Compound** | **Salinity (%)** | | | | |  | **Temperature (℃)** | | |
| --- | --- | --- | --- | --- | --- | --- | --- | --- | --- |
|  | **0** | **0.1** | **0.2** | **0.3** | **0.4** |  | **20** | **25** | **30** |
| **PFBA** | 0.66 | 0.68 | 0.69 | 0.71 | 0.70 |  | 0.64 | 0.67 | 0.70 |
| **PFHpA** | 0.30 | 0.37 | 0.35 | 0.36 | 0.42 |  | 0.34 | 0.34 | 0.37 |
| **PFOA** | 0.23 | 0.23 | 0.22 | 0.26 | 0.26 |  | 0.28 | 0.30 | 0.33 |
| **PFDoA** | 0.03 | 0.04 | 0.03 | 0.04 | 0.05 |  | 0.03 | 0.03 | 0.04 |

Table S5The transfer factors for each of the PFCAs at different exposure salinity and temperature conditions

| **Compound** | **Salinity (%)** | | | | |  | **Temperature (℃)** | | |
| --- | --- | --- | --- | --- | --- | --- | --- | --- | --- |
|  | **0** | **0.1** | **0.2** | **0.3** | **0.4** |  | **20** | **25** | **30** |
| **PFBA** | 0.85 | 1.06 | 1.34 | 2.00 | 2.61 |  | 0.73 | 0.98 | 1.21 |
| **PFHpA** | 0.46 | 0.82 | 1.12 | 1.39 | 2.10 |  | 0.36 | 0.61 | 0.89 |
| **PFOA** | 0.43 | 0.76 | 1.01 | 1.49 | 2.09 |  | 0.29 | 0.56 | 0.73 |
| **PFDoA** | 0.33 | 0.67 | 0.77 | 0.94 | 1.59 |  | 0.28 | 0.46 | 0.64 |
